# A Deep Learning Approach for Automatic Segmentation during Daily MRI-Linac Radiotherapy of Glioblastoma

**DOI:** 10.3390/cancers15215241

**Published:** 2023-10-31

**Authors:** Adrian L. Breto, Kaylie Cullison, Evangelia I. Zacharaki, Veronica Wallaengen, Danilo Maziero, Kolton Jones, Alessandro Valderrama, Macarena I. de la Fuente, Jessica Meshman, Gregory A. Azzam, John C. Ford, Radka Stoyanova, Eric A. Mellon

**Affiliations:** 1 Department of Radiation Oncology, Sylvester Comprehensive Cancer Center, Miller School of Medicine, University of Miami, Miami, FL 33136, USA; a.breto@umiami.edu (A.L.B.); kcullison@med.miami.edu (K.C.); rstoyanova@med.miami.edu (R.S.); 2 Department of Radiation Medicine & Applied Sciences, UC San Diego Health, La Jolla, CA 92093, USA; 3 West Physics, Atlanta, GA 30339, USA; 4 Department of Neurology, Sylvester Comprehensive Cancer Center, Miller School of Medicine, University of Miami, Miami, FL 33136, USA

**Keywords:** glioblastoma, auto-segmentation, MRI-linac, radiation therapy, deep learning, MRI

## Abstract

**Simple Summary:**

Current auto-segmentation methods for glioblastoma utilize mainly pre-operative 1.5T and 3T MRI. The first commercial MRI-linear accelerator (linac) radiation treatment platform acquires low-field (0.35T) post-operative MRI at the delivery of each treatment. This study presents the first automatic brain lesion segmentation network developed for MRI-linac to track tumor changes during radiotherapy. The tumors and resection cavities are automatically segmented using a deep learning network, allowing for daily monitoring of tumor volume changes, creation of tools necessary for adaptive radiotherapy of glioblastoma, and providing MRI regions of interest for further analyses that may discover prognostic markers.

**Abstract:**

Glioblastoma changes during chemoradiotherapy are inferred from high-field MRI before and after treatment but are rarely investigated during radiotherapy. The purpose of this study was to develop a deep learning network to automatically segment glioblastoma tumors on daily treatment set-up scans from the first glioblastoma patients treated on MRI-linac. Glioblastoma patients were prospectively imaged daily during chemoradiotherapy on 0.35T MRI-linac. Tumor and edema (tumor lesion) and resection cavity kinetics throughout the treatment were manually segmented on these daily MRI. Utilizing a convolutional neural network, an automatic segmentation deep learning network was built. A nine-fold cross-validation schema was used to train the network using 80:10:10 for training, validation, and testing. Thirty-six glioblastoma patients were imaged pre-treatment and 30 times during radiotherapy (*n* = 31 volumes, total of 930 MRIs). The average tumor lesion and resection cavity volumes were 94.56 ± 64.68 cc and 72.44 ± 35.08 cc, respectively. The average Dice similarity coefficient between manual and auto-segmentation for tumor lesion and resection cavity across all patients was 0.67 and 0.84, respectively. This is the first brain lesion segmentation network developed for MRI-linac. The network performed comparably to the only other published network for auto-segmentation of post-operative glioblastoma lesions. Segmented volumes can be utilized for adaptive radiotherapy and propagated across multiple MRI contrasts to create a prognostic model for glioblastoma based on multiparametric MRI.

## 1. Introduction

Glioblastoma is the most common and aggressive primary brain tumor in adults with a median overall survival of only 15 months [1,2]. The standard of care consists of tumor biopsy or surgical resection followed by six weeks of radiation therapy (RT) and concurrent temozolomide chemotherapy, and then continued temozolomide post-RT. Traditionally, glioblastoma RT is delivered through standard linear accelerators (linacs) that utilize cone-beam CT for daily treatment set-up. Although sufficient for patient positioning, soft tissue inside the skull is not visible on these scans, so tumor monitoring during treatment is not possible without additional imaging. Patients undergoing RT on standard linacs typically receive a high-field treatment planning MRI approximately 1–2 weeks before beginning RT, followed by MRI obtained approximately one month after radiation ends, which reveals tumor growth based on gadolinium T1 post-contrast imaging in up to 49% of patients compared to their pre-RT MRI [3]. Despite this, it was previously thought that glioblastomas did not change significantly during treatment, so adaptive RT is not common in glioblastoma management. Instead, the RT treatment field (typically gross disease plus a volumetric expansion—2 cm for glioblastoma [4,5]) is determined using the pre-RT MRI.

Although it is possible to obtain standalone high-field MRI during RT to investigate tumor anatomy during treatment, there is a feasibility limitation of how often this imaging can be done. Even centers with robust MRI research groups only tend to perform standalone high-field brain MRI at 1–2 timepoints during RT [6,7,8]. Previous feasibility limitations of frequent MRI during treatment are obviated using MRI-linac, since patients receive MRI as part of standard daily treatment set-up instead of cone-beam CT. This is the first time that we are able to obtain daily MRI for glioblastoma patients, which allows for detection of volumetric changes during treatment and other physiologic changes that may be prognostic. Uniquely, our center has been treating glioblastoma patients with MRI-linac since 2017, the first in the world to do so.

In addition to growth seen between pre- and post-RT scans, changing glioblastoma anatomy has recently been observed during MRI-guided RT, whether that is changing tumor or changing resection cavity anatomy [9]. There is rationale to monitor these anatomic changes during treatment: For shrinking resection cavities and tumors, there is potential to adapt radiation targets to the shrinking field, sparing heathy tissue from unnecessary dosing [10,11]. For tumors that grow significantly during treatment, adaptation could assist with missing disease outside of typical treatment margins that are based on older pattern of failure studies [4].

In addition to detecting anatomic changes, there is potential for MRI during treatment to monitor physiologic tumor changes that may have prognostic value. Tumor growth seen on post-RT MRI can be due to either: (1) neovascularization and inflammation from a positive treatment response termed pseudoprogression (median overall survival 38 months), or (2) proliferating cancer cells not responding to therapy termed true progression (median overall survival 10 months) [12,13,14]. Currently, true and pseudoprogression can only be deciphered from one another 3–6 months post-RT using serial MRI and corresponding clinical symptoms [15], although developing early imaging and biomarkers for outcome prediction is an area of intensive research [16]. If true progression could be distinguished from pseudoprogression earlier, there is potential for physiological adaptive (radio)therapy [17] based on predicted poor response—i.e., increased radiation dose, earlier second line therapies, etc., for those predicted to go on to have true progression.

The first step necessary to make possible anatomic and physiologic adaptive therapy for glioblastoma is to segment, or contour, the tumor regions of interest (ROI) on MRI. Manual contouring is time-intensive, requiring lesion segmentation on multiple axial slices on each of the thirty treatment images—30 min per scan, sometimes up to an hour for complicated tumors. Multiplied by 31 timepoints (1 pre-RT and 30 fractions), this is 15.5–31 h of labor per patient. Although software packages and networks already exist for automatic contouring of brain structures on MRI, almost all utilize pre-operative MRI (normal brain structures) [18,19,20], and although one published study exists for post-operative auto-segmentation of glioblastoma, it is on high-field MRI [21]. One of two current commercially available MRI-linacs is low-field MRI, which has its own unique challenges. Recently published work has shown the success of auto-segmentation for low-field MRI-linac [22,23,24], but these have largely focused on gastrointestinal and genitourinary anatomy. Combine the lack of networks specifically for low-field brain segmentation with the fact that post-operative images pose their own separate set of challenges since brain structure anatomy is altered by surgery, and this leaves an unmet need: no software or network currently exists for auto-segmentation of brain lesions on low-field, post-operative MRI (which constitutes our center’s novel dataset). Without such a network, adaptive RT for glioblastoma, either based on significant anatomic changes or underlying tumor physiology, is not feasible.

We sought to fill this unmet need by creating a network capable of auto-segmenting glioblastoma tumors on low-field, post-operative MRI from MRI-linac. Here, an integrated approach for simultaneous segmentation of both tumor and resection cavities on daily MRI-linac images using a deep neural network is presented. Mask Region-Based Convolutional Neural Network (R-CNN) was selected as the network because it has shown promise in the auto-segmentation of pelvic organs from the same low-field MRI-linac system utilized in this study [24]. The proposed network automatically generates ROI and determines the segmented volumes. The automatic segmentation takes 3 min on current typical computer hardware. Even in the scenario where the auto-segmented contours need additional physician/manual editing, the procedure will still be substantially shorter and less laborious than manual processing, while also being nearly rater-independent. These auto-segmented ROI will allow for real-time volumetric monitoring of tumor anatomy, create tools necessary to make adaptive RT for glioblastoma feasible, and constitutes the first step in creating an automated MRI analysis pipeline for identifying imaging markers that may be predictive of true vs. pseudoprogression in glioblastoma patients during treatment, which is 4–6 months earlier than current methods [25].

In this paper, we will discuss in Materials and Methods: our study patient characteristics and RT overview, MRI-linac imaging acquisition and manual tumor delineation definitions, and the proposed deep learning network and parameters. In Results, we present average Dice similarity coefficients between the manual and auto-segmented contours, which describes the spatial overlap between two contours. We also show four examples of manual vs. auto-segmented tumors, including manual and auto-segmented volume curves for each. Finally, in the Discussion, we touch on the relevant related literature, comparing our study to others where appropriate, the clinical and research applications of the proposed auto-segmentation network, study limitations, and future directions.

## 2. Materials and Methods

### 2.1. Study Patients and MRI-Linac Treatment

Glioblastoma patients from two single-institution prospective, Institutional Review Board-approved protocols (protocol number 20190678, date of approval 9 December 2019, and protocol number 20160817, date of approval 17 April 2017) underwent radiotherapy on 0.35T MRI-linac (ViewRay MRIdian, Cleveland, OH, USA). Glioblastoma was defined using the World Health Organization (WHO) 2016 criteria and also included patients with “diffuse astrocytic glioma, Isocitrate Dehydrogenase I (IDH)-wildtype, with molecular features of glioblastoma, WHO grade IV” per the Consortium to Inform Molecular and Practical Approaches to CNS Tumor Taxonomy (cIMPACT-NOW) Update 3 criteria [26]. Patients either received surgical resection or biopsy prior to treatment. Patient characteristics are summarized in Table 1.

After surgery, patients underwent a 1.5T or 3T pre-treatment RT planning MRI including gadolinium contrast-enhanced T1-weighted imaging, T2-weighted imaging, FLAIR, and additional sequences. Treatment planning for each patient was also performed at that time on MRI-linac including an MRI-compatible thermoplastic mask immobilization system. A maximum of 30 daily treatment set-up MRIs from MRI-linac were acquired for each patient, for a maximum of 31 MRI-linac time points per patient. Patients received a total dose of 60 Gy over 30 treatment fractions using MRI-linac concurrent with temozolomide 75 mg/m^2^.

### 2.2. Imaging Acquisition and Tumor Delineation

The MRI-linac was equipped with a 0.35T MRI (Siemens Avanto, version Syngo MR B17, IDEA version VB19, Siemens Medical Solutions, Erlangen, Germany). The MRI-linac includes a balanced steady-state free precession (bSSFP) pulse sequence as the daily treatment set-up scan [27]. The bSSFP is commonly used in relatively low-field MRI acquisition due to its capability of providing an increased signal-to-noise ratio with higher temporal resolution [28,29]. The signal originating from the bSSFP sequence is also known for its mixed composition that is weighted by both T1 and T2. However, the use of high flip angles produces fluid-bright, predominantly T2-weighting as implemented on this 0.35T system [30]. The bSSFP images were acquired with the following parameters: TE (time to echo)/TR (time to repetition) = 1.92 ms/3.84 ms, flip angle = 60°, acquisition matrix = 266 × 288 × 266, Voxel size 1.5 mm × 1.5 mm × 1.5 mm (3D-acquisition), bandwidth 532 Hz/Px, acquisition time = 2 min and 8 s.

Tumor lesion and resection cavity were manually contoured on each available bSSFP MRI on MIM Version 7.2.3 (MIM Software Inc., Cleveland, OH, USA) by imaging experts (ALB, KC, RS) with combined experience of more than 25 years and reviewed by an attending radiation oncologist (EAM). Tumor lesion includes the glioblastoma tumor plus any surrounding edema, which is sometimes difficult to separate from true tumor on T2-weighted images.

### 2.3. Deep Learning Network for Auto-Segmentation of Tumor and Resection Cavity

The pipeline for MRI pre-processing: contours for network training, validation, and testing, as well as the Mask R-CNN implementation for application to bSSFP images were previously described [24]. Briefly, Mask R-CNN works by segmenting objects based on pixel changes, and then provides a binary mask of the ROI as well as a prediction of the mask class [31]. For our study, Mask R-CNN was implemented in Tensorflow [32] and Keras [33]. Raw image intensities were normalized to the interval of [0, 1] and, from the whole MRI volume of data, only axial slices with at least one manual contour were used for training. Initially, the network weights were loaded from a trained ImageΝet model [34]. The network’s training parameters were configured as described by Johnson [35]. The optimization algorithm selected is Stochastic Gradient Descent (SGD) with a learning rate α = 0.01, momentum of 0.9, and decay of 10^−6^. The training was performed with a batch size of 18.

The network masks were generated at the original resolution of the input, single axial images, as described above. A nine-fold cross-validation schema (80:10:10 for training, validation, and testing) was used to train and evaluate the network. Each fold was trained independently, with the test set unseen by the resulting network until training was completed.

Three-dimensional (3D) masks were obtained through stacking the individual two-dimensional (2D) slices segmented via Mask R-CNN across the inferior-superior axis. The obtained 3D masks were smoothed using recursive Gaussian filtering and intensity thresholding, followed by binary morphological closing to remove small holes and tube-like structures in the interior and at the boundaries of the 3D shapes. The performance of the method was assessed via the Dice similarity coefficient (DSC) [36] between the 3D manual and Mask R-CNN automated masks on a per-timepoint image basis. Small ROI < 2 cc were excluded from this DSC analysis.

## 3. Results

Thirty-six patients with glioblastoma were included in this analysis. From this cohort, 12 patients underwent gross total resection, 14 underwent sub-total resection, and 10 patients underwent biopsy only. Defined on the MRI-linac bSSFP planning scan, 8 patients had visible resection cavity only, 16 had a mix of both resection cavity and tumor lesion, and 12 had tumor lesion only. These numbers differ from the gross total resection, sub-total resection, and biopsy numbers for a variety of reasons. Some patients had rapid early progression with tumor growth in between surgery and the start of radiation [37]. Some patients had non-enhancing tumor left post-surgery despite being classified as gross total resection. Finally, some patients with sub-total resection had resection cavity shrinkage between surgery and the start of radiation, leaving only tumor lesion visible on their bSSFP scans. Additional details of the patients’ clinical parameters are given in Table 1.

The nine-fold cross-validation schema was realized by eight patients per-fold set aside to serve as a validation and training set (four patients per set, respectively). The remaining 28 patients were submitted to the network as a training set. The total dataset was preprocessed into a total of 37,751 axial slices, roughly 1179 per patient. The average volume for tumor lesion and resection cavity was 94.56 ± 64.68 cc and 72.44 ± 35.08 cc, respectively. The average DSC for tumor lesion and resection cavity across all patients was 0.67 and 0.84, respectively. For patients with tumor lesion only, the average DSC was 0.67. For patients with resection cavity only, the average DSC was 0.89. For patients with mixed (both tumor lesion and resection cavity classes), the average DSC was 0.67 for tumor lesion and 0.81 for resection cavity. Additional details are shown in Table 2, where small ROI (<2 cc) were excluded from analysis due to the network’s difficulty with lesions this small.

**Figure 1:**
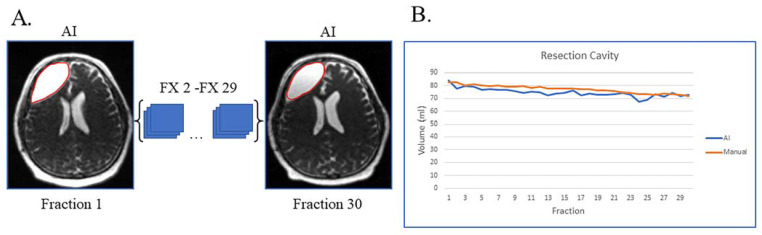
Gross total resection with shrinking resection cavity and no evidence of disease progression on post-treatment MRI. (**A**) Auto-segmented contours of resection cavity (red) at the first and last treatment fractions (FX) shown on MRI-linac bSSFP. (**B**) Volumes derived from AI (blue) and manual segmentation (orange) across treatment fractions.

Figure 1A shows an example of a patient with resection cavity only. This case is simple, as the patient had a gross total resection of their disease with no visible tumor lesion left on their MRI-linac bSSFP scans. This patient had subtle resection cavity shrinkage during treatment, picked up by both the deep learning artificial intelligence (AI) and manual segmentations (Figure 1B), and the AI volumes over the 30 treatment fractions track closely with the manually segmented volumes.

**Figure 2:**
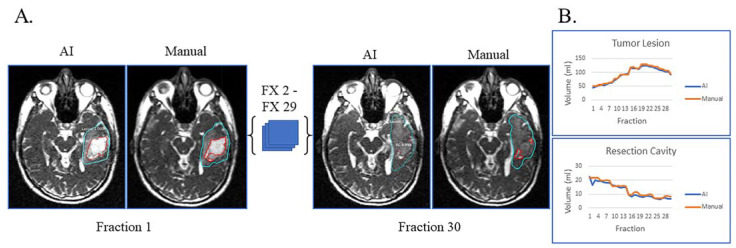
Sub-total resection with resection cavity and tumor lesion. True progression of tumor lesion was observed during the majority of treatment and later confirmed. A decrease in tumor lesion (likely edema) is seen towards the end of treatment when dexamethasone was started due to progressive headache. (**A**) Auto-segmentation (left) and manual (right) contours of tumor lesion (cyan) and resection cavity (red) at the first and last treatment fractions (FX) shown on MRI-linac bSSFP. (**B**) Volumes derived from AI (blue) and manual segmentation (orange) across treatment fractions for tumor lesion and resection cavity.

Figure 2A displays the automatic and manual contours of a patient with both resection cavity and tumor lesion after a sub-total resection. This patient had true progression, defined by tumor growth on high-field-MRI one month after the completion of RT with progressive worsening of disease on serial high-field MRI over the next six months that did not stabilize or improve. In addition to post-RT progression, this patient had progressive growth of their tumor during most of their treatment and mild resection cavity shrinkage. Of note, there is a volume decrease in tumor lesion volume starting around fraction 22, typically not seen with true progression (Figure 2B). However, this patient tapered off dexamethasone steroids prior to the beginning of RT but started back on dexamethasone around fraction 22 due to worsening symptoms, which could explain the decrease in tumor lesion volume seen towards the end of treatment. Despite this complexity, the AI-derived volumes over the 30 treatment fractions track closely with the manual ones for both resection cavity and tumor lesion.

**Figure 3:**
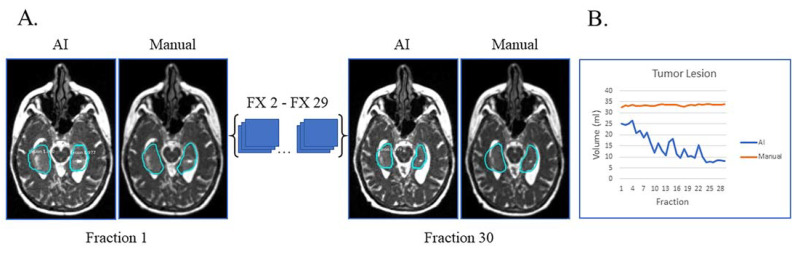
Biopsy only with tumor lesion without progression during RT and no progression on post-treatment MRI. (**A**) Auto-segmentation (left) and manual (right) contours of tumor lesion (cyan) at the first and last treatment fractions (FX) shown on MRI-linac bSSFP. (**B**) Volumes derived from AI (blue) and manual segmentation (orange) across treatment fractions demonstrate subtle shrinkage not appreciated by manual contouring but apparent in retrospect.

In Figure 3A, the patient had tumor lesion only following a biopsy. This is an example of a case where the AI volumes deviate from the manual volumes: the trajectory from the manual contours shows little to no volume change during treatment while the AI contours show a decreasing volume trend (Figure 3B). On re-review of this subject’s imaging, there is subtle visual tumor lesion shrinkage over time, consistent with the AI contours. This case points out that there can be errors in manual contours, in particular when reviewing small daily or weekly changes. In this subject’s case, it is fair to say that the network performed a superior job to the manual contours.

**Figure 4:**
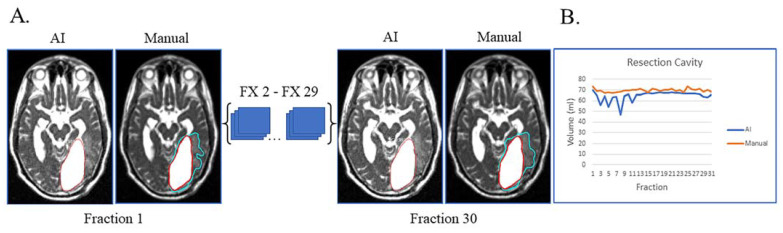
Case of network failure due to unusual anatomy. Patient had sub-total resection with resection cavity and tumor lesion with no progression of disease during and immediately after treatment. (**A**) Auto-segmentation (left) and manual (right) contours of tumor lesion (cyan) and resection cavity (red) at the first and last treatment fractions (FX) shown on MRI-linac bSSFP. The large resection cavity communicated with ventricle, which led to the network’s failure to detect an obvious tumor lesion surrounding resection cavity at all timepoints. (**B**) Still, AI-derived volumes (blue) compared to manually segmented volumes (orange) for resection cavity across treatment.

Lastly, unusual findings outside the training of a dataset can cause AI failures, as shown in Figure 4. This patient had sub-total resection of disease with resection cavity and tumor lesion. After surgery, the left occipital ventricular horn became trapped by the central tumor with large expansion of the ventricle and surrounding edema (Figure 4A). This case highlights that anatomic changes seen during RT can be unique/unexpected, which can affect the network’s ability to segment. Despite these challenges, the network was still able to segment resection cavity with relatively high accuracy (Figure 4B).

## 4. Discussion

Deep learning approaches have been widely used for brain segmentation, and they have shown promise for glioblastoma auto-segmentation. The Multimodal Brain Tumor Segmentation (BraTS) challenge provides a multi-institutional glioma MRI dataset with four contrasts (T1-weighted, T1 contrast-enhanced (T1ce), T2-weighted, and FLAIR) and ground truth manual contours of tumor sub-regions [19]. The nnU-Net [38] was the top-performing network of the challenge in 2021 with a DSC of 0.9275. Although highly successful models of auto-segmentation, the imaging data utilized in BraTS pose three major differences from our data: (1) they are acquired on a high-field MRI (mostly 3T, with some acquired on 1.5T); (2) the MRI scans are all pre-operative; and (3) they use multiple MRI contrasts. For the current study’s dataset: (1) the MRI-linac utilized is low-field MRI; (2) all patient scans analyzed are post-operative, except in the minority of patients that have biopsy only prior to beginning RT; and (3) a single contrast is used from the MRI-linac as it is available for every patient treated without added daily acquisition time.

Eliminating the comparison of networks trained on pre- and post-operative images, Gazit et al. analyzed T1ce scans from 340 patients and developed a method for segmentation of post-operative glioblastoma lesions [21]. Their results—average DSC for resection cavity and T1ce enhancing lesions of 0.71 and 0.68, respectively—compare favorably with the results from this study—average DSC for resection cavity and tumor lesion 0.84 and 0.67, respectively. This is regardless of the smaller number of patients used for network training and the lack of T1ce scans used in our present study. The relatively robust performance of our network is due in part to the utilization of Mask R-CNN [24]. Its backbone, the ResNet50 network, is pre-trained with images from the ImageNet database, containing over 14 million images. Instead of starting the training from scratch, the trained “weights” of ImageNet are used by default, facilitating transfer learning [39]. This allows the network to be trained satisfactorily on new datasets with few examples. The other contributing factor is that the MRI data were acquired on the same instrument with the same sequences; the lack of variability in the data acquisition due to different vendors and sequences contribute to the performance of the network [40,41].

There are immediately translatable clinical applications of the proposed auto-segmentation network, such as to provide automatic tumor volume tracking for the clinical team. This could be useful to radiation oncology physicians, who may not be looking at the RT set-up MRIs of patients every day, or for treatment of patients where the daily MRI changes are subtle to the naked eye because the tumor is growing or changing slowly over time (such as was the case in Figure 3). A system implementation of volume change alerts would allow the clinical team to review and adapt the radiation field to the evolving tumor anatomy in real time. Additionally, the auto-segmentation could be used as a starting point (editing as needed) for physicians as the gross tumor volume for the adaptive RT plan. As mentioned in the introduction, this would save time, since typically the clinical team has to manually re-contour a tumor for any adaptive RT case, which is a limiting factor in the feasibility of adaptive RT in general [42].

A central limitation of this study is the lack of serial contrast imaging. Typically, the amount of gadolinium enhancement on MRI is the primary metric used to evaluate glioblastoma evolution by criteria like Response Assessment in Neuro-Oncology (RANO) [43] and delineation of other tumor types [44]. Thus, a challenge to the MRI-linac community is when and how often to administer contrast during MRI-guided RT. Our center has found frequency of gadolinium administration and gadolinium deposition with repeated contrast administrations to be a significant concern among patients [45]. These concerns, although valid, have not been shown to have significant clinical consequences, and repeated gadolinium-based contrasts is considered safe in patients with normal renal function [46], but the U.S. Food & Drug Administration still advises to “minimize repeated [gadolinium contrast] imaging studies when possible, particularly [for] closely spaced MRI studies” [45]. However, rapid tumor growth during RT is visible on non-contrast MRI-linac images [17], including the T2-weighted bSSFP images utilized in this study. This could trigger gadolinium contrast-enhanced MRI on either MRI-linac [47] or standalone MRI in select patients. Other limitations include the small number of patients, the network’s low performance on small lesions (<2 cc), and the potential limited generalizability of the current model outside of the 0.35T MRI-linac system.

Future directions include the use of the network-created contours for research analyses seeking to identify prognostic imaging markers. This includes radiomics and delta-radiomics, parametric response mapping, pattern analysis of volume trajectories, and other quantitative MRI methods [17,48], many of which are ongoing at our center now. These methods can identify prognostic markers based on tumor physiology, which would be used to adapt/intensify treatment for patients with predicted poor outcome (true progression). Many studies have previously explored the topic of predicting response based on quantitative MRI metrics, but a general limitation has been the reproducibility of these results at different centers with differing MRI field strengths, manufacturers, systems, and analysis methods [49]. A benefit of applying this type of research to MRI-linac is that the MRI is identical across every site with the same manufactured machine, which increases the likelihood of reproducible results, as well as provides independent external cohorts with which to validate methods.

Other future directions include improving this network with additional patients’ imaging, as well as comparing the results from the current proposed network (Mask R-CNN) with another network (nnU-Net) to see which method is superior. This is ongoing currently at our center. Other ideas are utilizing machine learning networks to generate additional low-field images to increase sample size [22], multi-contrast MRI-linac imaging at 0.35T [17,47,50], transfer learning [39] of the current network with these additional MRI contrasts for a more robust network, and developing patient-specific models that improve additional contours using the previous timepoints’ edited contours as fine-tuning [51].

## 5. Conclusions

Here we present a deep learning approach for automatic segmentation of glioblastoma on daily, post-operative, low-field MRI. This is the first automatic brain lesion segmentation network developed for MRI-linac. The network performed well overall with average DSC of 0.67 for tumor lesion and 0.84 for resection cavity, comparable to the only other published network for auto-segmentation of post-operative glioblastoma lesions (average DSC of 0.68 for lesion and 0.71 for resection cavity). Additional deep learning approaches and/or MRI contrasts could potentially improve future auto-segmentation efforts and DSC values. There are various exciting applications for the proposed network, including: the first available system for automatic glioblastoma volume tracking during radiation therapy; delivering contours for the investigation of imaging markers prognostic of glioblastoma outcome (true and pseudoprogression); and providing key items for making early anatomic and/or physiologic adaptive (radio)therapy feasible for glioblastoma, with the goal of improving overall survival times.

## Figures and Tables

**Table 1:** Patient characteristics.

Variable	N (%)
Age, Median (Range)	60 (22–77)
Sex	
Female	12 (33%)
Male	24 (67%)
Resection type	
Gross total	12 (33%)
Sub-total	14 (39%)
Biopsy	10 (28%)
O6-Methylguanine-DNA Methyl-Transferase (MGMT) Status	
Hypermethylated	15 (42%)
Non-Hypermethylated	17 (47%)
Unknown	4 (11%)
Isocitrate Dehydrogenase I (IDH) Status	
Mutant	6 (17%)
Wild Type	30 (83%)

**Table 2:** Summary of Dice similarity coefficient (DSC) values from the deep learning network.

	Global		TL		RC		Mixed (Both RC and TL)	
	*n*	DSC (Mean ± σ)	*n*	DSC (Mean ± σ)	*n*	DSC (Mean ± σ)	*n*	DSC (Mean ± σ)
**Tumor Lesion (TL)**	20	0.67 ± 0.2	15	0.67 ± 0.17	0		5	0.67 ± 0.24
**Resection Cavity (RC)**	12	0.84 ± 0.18	0		7	0.89 ± 0.06	5	0.81 ± 0.14

## Data Availability

The data presented in this study will be submitted to The Cancer Imaging Archive pending acceptance of this manuscript.
